# Involvement of the Cohesin Cofactor PDS5 (SPO76) During Meiosis and DNA Repair in *Arabidopsis thaliana*

**DOI:** 10.3389/fpls.2015.01034

**Published:** 2015-12-01

**Authors:** Mónica Pradillo, Alexander Knoll, Cecilia Oliver, Javier Varas, Eduardo Corredor, Holger Puchta, Juan L. Santos

**Affiliations:** ^1^Departamento de Genética, Facultad de Biología, Universidad ComplutenseMadrid, Spain; ^2^Botanical Institute II, Karlsruhe Institute of TechnologyKarlsruhe, Germany

**Keywords:** *Arabidopsis thaliana*, cohesin cofactor, DNA repair, homologous recombination, Meiosis, PDS5, SPO76

## Abstract

Maintenance and precise regulation of sister chromatid cohesion is essential for faithful chromosome segregation during mitosis and meiosis. Cohesin cofactors contribute to cohesin dynamics and interact with cohesin complexes during cell cycle. One of these, PDS5, also known as SPO76, is essential during mitosis and meiosis in several organisms and also plays a role in DNA repair. In yeast, the complex Wapl-Pds5 controls cohesion maintenance and colocalizes with cohesin complexes into chromosomes. In *Arabidopsis*, AtWAPL proteins are essential during meiosis, however, the role of AtPDS5 remains to be ascertained. Here we have isolated mutants for each of the five *AtPDS5* genes (A–E) and obtained, after different crosses between them, double, triple, and even quadruple mutants (*Atpds5a Atpds5b Atpds5c Atpds5e*). Depletion of AtPDS5 proteins has a weak impact on meiosis, but leads to severe effects on development, fertility, somatic homologous recombination (HR) and DNA repair. Furthermore, this cohesin cofactor could be important for the function of the AtSMC5/AtSMC6 complex. Contrarily to its function in other species, our results suggest that AtPDS5 is dispensable during the meiotic division of *Arabidopsis*, although it plays an important role in DNA repair by HR.

## Introduction

Cohesin is a ring-shaped protein complex which holds sister chromatids together to prevent their separation prior to anaphase. Genes coding cohesin subunits are evolutionarily conserved as are the general mechanism of action of the corresponding proteins. The cohesin complex is formed by four core components, a heterodimer of Structural Maintenance of Chromosome proteins (SMC1 and SMC3) and two non-SMC proteins. The non-SMC proteins are SCC3 (SA1-SA2/STAG1-STAG2), and a member of the conserved α-kleisin family: SCC1 (Mcd1/RAD21, known as SYN proteins in *Arabidopsis thaliana*, see below) (Nasmyth and Haering, 2009). In germ cells, meiosis-specific cohesin subunits have been characterized: SMC1β and STAG3, encoded by *SCC3* homologues, and the α-kleisin REC8 (SYN1 in *A. thaliana*) (Suja and Barbero, 2009). In addition to *RAD21* and *REC8*, a third α-kleisin gene, named *RAD21L*, specifically expressed in meiotic cells, has been identified in vertebrates (Gutiérrez-Caballero et al., 2011; Herrán et al., 2011; Ishiguro et al., 2011; Lee and Hirano, 2011). The cohesin protein complex is not only essential for sister chromatid cohesion, but it is also involved in chromosome condensation, gene expression, development, DNA repair and HR (Hirano, 2006; Dorsett, 2007; Onn et al., 2008; Barbero, 2009; Aragon et al., 2013).

In addition to proteins mentioned above, several non-cohesin accessory proteins contribute to cohesion regulation, although they are not considered to be components of the canonical cohesin complex (Nasmyth, 2011). The adherin complex SCC2–SCC4 is involved in cohesin loading during early G1 in vertebrate cells and late G1 in yeast (Ciosk et al., 2000; Watrin et al., 2006; Hu et al., 2011). The establishment of cohesion also requires SMC3 acetylation by Eco1/Ctf7p in yeast and ESCO1 and ESCO2 in human cells (Skibbens et al., 1999; Tóth et al., 1999; Hou and Zou, 2005). In mammalian cells, SMC3 acetylation is necessary for the recruitment of Sororin to chromatin-bound cohesin complexes, a protein needed for maintaining cohesion during G2/M (Rankin et al., 2005). Sororin stabilizes cohesin on DNA by competing with the cohesin release factor WAPL (Gandhi et al., 2006; Kueng et al., 2006; Nishiyama et al., 2010). WAPL is a negative regulator of cohesion that interacts directly with RAD21 and SA/STAG cohesin subunits and colocalizes with the axial element protein SYCP3 in mouse spermatocytes and oocytes (Kuroda et al., 2005; Zhang et al., 2008). WAPL forms a complex with PDS5 (Precocious Dissociation of Sisters 5), a large HEAT-repeat-containing protein that also interacts with the cohesin complex by binding α-kleisin (Neuwald and Hirano, 2000; Kueng et al., 2006; Shintomi and Hirano, 2009). Sororin also directly associates with PDS5 and thereby dissociates WAPL from PDS5, at least *in vitro*, implying that Sororin antagonizes WAPL by changing its interaction with PDS5 (Nishiyama et al., 2010). The interaction of PDS5 with either Sororin or WAPL could explain why this protein has both positive and negative effects on cohesion association.

Regarding the PDS5 function, there are also important differences among organisms. In *Saccharomyces cerevisiae*, Pds5p is essential for viability and is required to maintain sister chromatid cohesion and chromosome condensation (Hartman et al., 2000; Panizza et al., 2000; Stead et al., 2003; Tong and Skibbens, 2014). A recent study has determined that Pds5 in yeast maintains cohesion, at least in part, by antagonizing the polySUMO-dependent degradation of cohesin (D’Ambrosio and Lavoie, 2014). BIMD, encoded by the homolog of *PDS5* in *Aspergillus nidulans*, has also an important function in maintaining cohesion. Mutations in the *BIMD* gene result in mitotic arrest at anaphase and an increased sensitivity to DNA damaging agents (Denison et al., 1993). In contrast, *pds5*-null strains are viable in *Schizosaccharomyces pombe*, but a *pds5Δ* mutation confers cohesion defects after prolonged arrest in G2/M and increased chromosome loss rates (Tanaka et al., 2001; Wang et al., 2002). *Xenopus* eggs extracts depleted of both *PDS5A* and *PDS5B* (there are two *PDS5* genes in vertebrates) display an abnormal level of cohesin on chromosomes and altered centromeric cohesion (Sumara et al., 2000; Losada et al., 2005). Other studies have demonstrated that knockout mice for either *PDS5A* or *PDS5B* die at the perinatal age with several developmental anomalies that resemble those found in humans with Cornelia de Lange syndrome (Zhang et al., 2007, 2009). Recently, an analysis of primary mouse embryonic fibroblasts lacking PDS5A, PDS5B, or both has revealed that they contribute to telomere and arm cohesion. In addition, PDS5B is specifically required for centromeric cohesion (Carretero et al., 2013).

The meiotic function of PDS5 was initially described in *Sordaria macrospora*, where it was named SPO76 (Moreau et al., 1985; Huynh et al., 1986). In this species SPO76 is needed for normal meiotic chromosome morphogenesis. The *spo76-1* mutant is defective in chromatid cohesion and chromosome compaction during prophase I, since chromatids are fully separated at diplotene. Meiotic recombination is also affected (van Heemst et al., 1999; Storlazzi et al., 2003). BIMD of *A. nidulans* is also required for cohesion and normal chromosome compactness during meiosis. However, in contrast to SPO76, it does not reveal defined axes during prophase I (van Heemst et al., 2001). In *Saccharomyces*, a meiosis-conditional *pds5* allele produces hypercondensed chromosomes and alterations in synapsis, DSB repair, and meiotic chromosome segregation (Jin et al., 2009). Cohesion defects were also observed during meiosis in *Caenorhabditis elegans elv-14*/*pds5* mutants. In this species, EVL-14 is not required for establishing sister chromatid cohesion but it is important for its maintenance (Wang et al., 2003).

In *A. thaliana*, single copy genes code for AtSMC1, AtSMC3, and AtSCC3. These proteins have been identified in both somatic and meiotic tissues (Liu et al., 2002; Chelysheva et al., 2005; Lam et al., 2005; Schubert, 2009). However, there are four *SCC1* homologues: *SYN1* (*DIF1*), *SYN2* (*RAD21.1*), *SYN3* (*RAD21.2*), and *SYN4* (*RAD21.3*). Homozygous knockout mutants of any of these genes are viable, probably because of the functional redundancy of them. SYN1 is needed for meiotic cohesion, whereas SYN2 and SYN4 are mitotic α-kleisins, with SYN2 also playing a role in DNA repair (Bai et al., 1999; Bhatt et al., 1999; Dong et al., 2001; Cai et al., 2003; da Costa-Nunes et al., 2006; Jiang et al., 2007; Yuan et al., 2012). See also Schubert (2009) for a detailed description of SMC complexes in plants. Regarding non-cohesin accessory genes, as in yeast a *Sororin* ortholog has not been identified in *A. thaliana*. Mutations in *AtCTF7* cause embryo lethality, since AtCTF7 is required for the establishment of sister chromatid cohesion and to avoid the premature dissociation of cohesin from chromosomes during meiosis (Jiang et al., 2010; Bolaños-Villegas et al., 2013; Singh et al., 2013). By contrast, plants without *AtWAPL* activity (there are two genes in *A. thaliana*) exhibit normal growth and development, but several defects during meiosis (De et al., 2014). Moreover, mutations in both *AtWAPL* genes suppress the lethality associated with inactivation of *AtCTF7*. The role of *AtPDS5* (there are five orthologs) and their possible involvement in *Arabidopsis* meiosis is still completely unknown. Here we report findings related to *AtPDS5* function, by analyzing the corresponding mutants. The results indicate that, contrary to *AtWAPL*, the absence of AtPDS5 causes growth defects, hypersensitivity to DNA repair and a drastic reduction in HR, but only subtle meiotic alterations.

## Materials and Methods

### Plant Material and Growth Conditions

*Arabidopsis thaliana* ecotype Columbia (0) was used for WT analysis. T-DNA lines corresponding to the five *AtPDS5* genes were the following: *AtPDS5A* (SALK_114556), *AtPDS5B* (SALK_092843), *AtPDS5C* (SALK_013481), *AtPDS5D* (SALK_133849), and *AtPDS5E* (SAIL_287_D07). They were obtained from the Nottingham *Arabidopsis* Stock Centre (NASC^1^). Plants were grown on a soil mixture of vermiculite and commercial soil (3:1) with a light cycle of 16 h alternating with 8 h of darkness, at 20°C and 70% humidity.

### *In Silico* Analysis

The program Clustal W2 was used for sequence alignment and to determine sequence identity between the proteins from different species (Larkin et al., 2007; McWilliam et al., 2013). The sequences were available in NCBI database. Scores for amino acid identity and similarity were retrieved from SIAS server^2^. The web-based system Genevestigator^®^ was used to obtain expression data with the experimental context variables anatomy and development (Hruz et al., 2008). The tool *Arabidopsis* Interactions Viewer from the BAR^3^ was used to determine AtPDS5 interacting proteins (Geisler-Lee et al., 2007).

### Molecular Characterization of *Atpds5* Mutants and *AtPDS5* Expression Analyses

Genotyping of the different T-DNA lines and expression analyses were performed as previously described by Pradillo et al. (2012). Details of the primers used are given in Supplementary Tables S1 and S2. In the real time PCR expression was normalized against 18S rRNA and *ACTIN2*, considering fold variation over a calibrator using the ΔΔ*C*_t_ method (Livak and Schmittgen, 2001). Three experimental replicates were performed, corresponding to at least two biological replicates.

### Cytological Procedures

Fixation, chromosome spread preparations of pollen mother cells (PMCs), fluorescence *in situ* hybridisation (FISH) and chiasma counts were carried out as described by Sánchez-Morán et al. (2001). Characteristics of 45S rDNA and 5S rDNA DNA probes are also provided in this reference.

Immunolocalization was performed by spreading as previously described (Armstrong et al., 2009). Primary antibodies used were: anti-AtZYP1 (rat; 1:500), anti-SYN1 (rabbit; 1:500), and anti-AtSMC3 (rabbit; 1:500) (Mercier et al., 2003; Higgins et al., 2005; Tiang, 2010).

Slides were observed using an Olympus BX-60 microscope equipped with an Olympus DP71 digital camera controlled by analysis software (Soft Imaging System). Images were analyzed and processed with Adobe Photoshop CS4.

### Genotoxicity Assays

Evaluation of hypersensitivity to γ-rays, and cisplatin [cis-diamminedichloroplatinum(II), CDDP, Sigma] were performed according to Oliver et al. (2014). The effects of these agents were evaluated 14 days after sowing. The mitomycin C (MMC, Duchefa Biochemie) test was carried out as previously described (Hartung et al., 2007). Briefly, surface-sterilized seeds of the tested lines were sown on plates containing solid germination medium (GM). After a week of growth, exactly 10 seedlings were placed into each well of six well plates containing 5 ml of liquid GM. After 1 day, different concentrations of MMC diluted in liquid GM were added for final concentrations of 5, 10, 15, and 20 μg/ml MMC. After a total of 2 weeks of growth in liquid medium, plantlets of each well were removed, briefly dried with a paper towel to remove excess liquid and then weighed on an analytical scale. Fresh weights of MMC-treated plants were set in relation to fresh weights of untreated plants of the same line. Statistical analyses were managed with the software SPSS Statistics 17.0.

### HR Assay

The HR assays were performed as described by Hartung et al. (2007). Briefly, fifty 7-day-old seedlings of mutant lines containing the *IC9* reporter construct (Molinier et al., 2004) were removed from plates containing solid GM and were transferred into halved petri dishes containing 10 ml liquid GM. After 1 day, bleomycin to the final concentration of 5 μg/ml was added if necessary. After a total of 8 days of growth in liquid GM, the seedlings were transferred into the staining solution (46.5 ml phosphate buffer pH 7, 1 ml of 5% sodium azide, 2.5 ml of 1% X-GlcA dissolved in DMF) for histological staining. Following 2 days incubation at 37°C in the staining solution, the plantlets were transferred into 70% ethanol at 60°C overnight for the extraction of plant pigments. Finally, the number of somatic recombination events was quantified by counting the number of blue sectors on plants using a binocular microscope.

## Results

### *Arabidopsis* Possesses Five *PDS5* Genes

**FIGURE 1:**
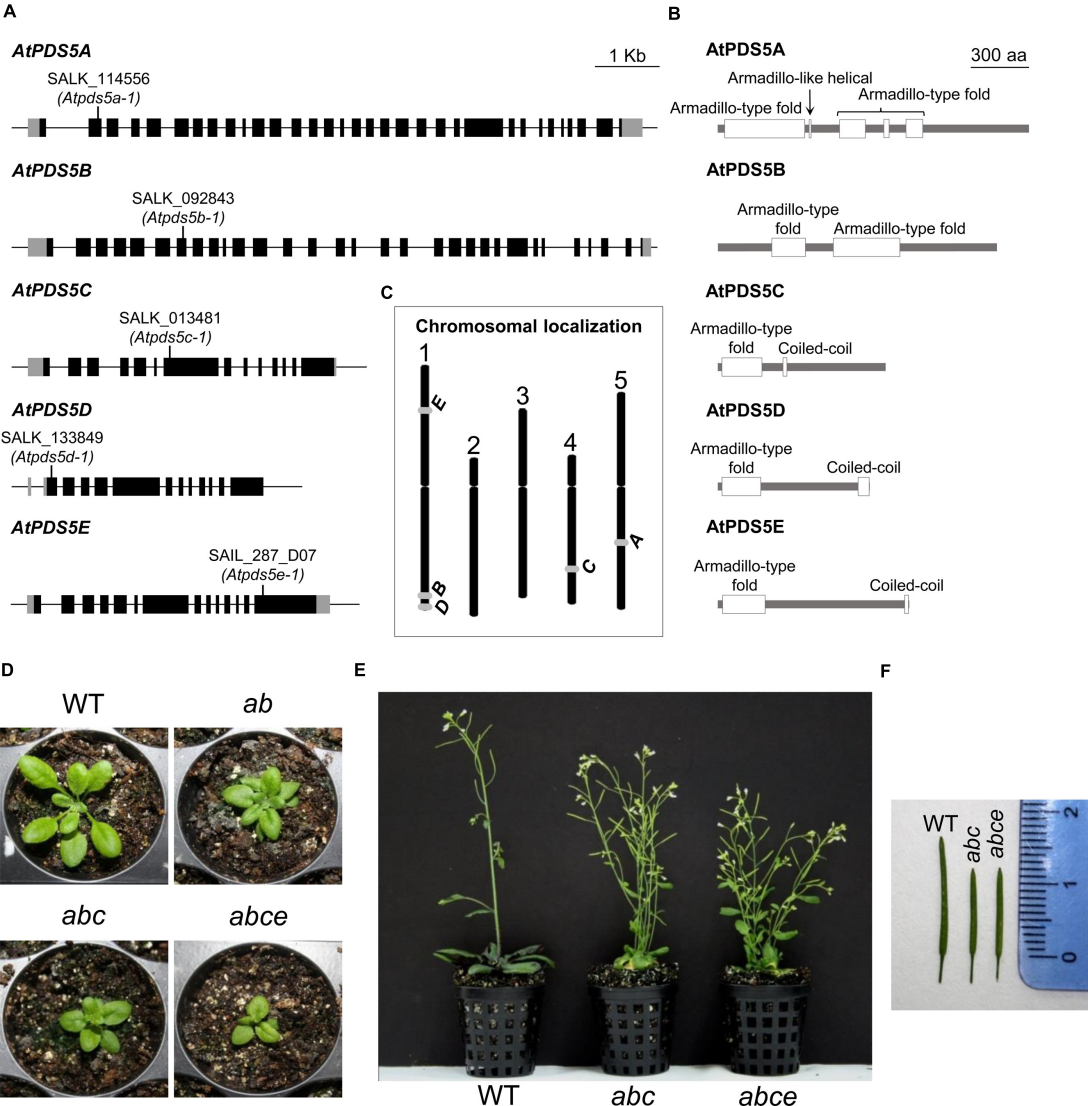
***AtPDS5* genes, AtPDS5 proteins and plant phenotypes of *Atpds5* mutants. (A)** An illustration of the exon/intron organization of *AtPDS5* genes. Exons are shown as black boxes and introns are shown as black lines. UTR regions are shown as gray boxes. Positions of T-DNA insertion sites of the different mutants are marked. **(B)** Schematic representation of the domains present in AtPDS5 proteins. **(C)** Chromosomal localization of the five *AtPDS5* orthologs. **(D)** Leaf rosette structure of 1 month-old plants imaged just before (WT, double mutant) or after (triple and quadruple mutants) bolting. **(E)** Six week-old WT, triple and quadruple mutant plants. **(F)** Siliques of WT, triple and quadruple mutant plants. *ab*: *Atpds5a Atpds5b*; *abc*: *Atpds5a Atpds5b Atpds5c*; *abce*: *Atpds5a Atpds5b Atpds5c Atpds5e*.

According to previous results and database searches of putative *PDS5* genes in the *Arabidopsis* genome, five candidates were identified (Mercier et al., 2001; **Figures 1A,C**). The protein that displays the highest identity and similarity to *S. macrospora* sequences (*SPO76*, 15% identity and 27% similarity), *A. nidulans* (*BIMD*, 15% identity and 26% similarity) and *Mus musculus* (*PDS5A*, 22% identity and 30% similarity; and *PDS5B*, 22% identity and 32% similarity) is encoded by At5g47690. For this reason we designated it as AtPDS5A. The remaining proteins were named according to their identity (ranging from 31 to 23%) and similarity (ranging from 39 to 20%) respect to AtPDS5A: AtPDS5B (At1g77600), AtPDS5C (At4g31880), AtPDS5D (At1g80810) and AtPDS5E (At1g15940). AtPDS5D and AtPDS5E are the most similar to each other (53%). As well as PDS5 proteins from other organisms, according to UniProt database, all AtPDS5 proteins contain an Armadillo-type fold domain in the or near to the N-terminus (UniProt Consortium, 2015; **Figure 1B**). This domain presents an extensive solvent-accessible surface that promotes interactions with proteins and nucleic acids.

### Characterization of *Atpds5* Mutants

To unravel the function of the *Arabidopsis PDS5* homologs, the NASC database was screened for lines containing a T-DNA insertion in the corresponding genes. Homozygous plants for the following lines were characterized: SALK_114556 (*Atpds5a-1*, T-DNA insertion located in the second exon), SALK_092843 (*Atpds5b-1*, T-DNA insertion located in the seventh exon), SALK_013481 (*Atpds5c-1*, T-DNA insertion located in the seventh exon), SALK_133849 (*Atpds5d-1*, T-DNA insertion located in the first exon), and SAIL_287_D07 (*Atpds5e-1*, T-DNA insertion located in the fourteenth exon) (**Figure 1A**). To simplify we have omitted the number corresponding to the allele designation throughout the text. To identify whether these lines generate full-length transcripts, RT-PCR was performed using cDNA from flower buds confirming the absence of a transcript spanning the T-DNA insertion (Supplementary Table S1). Furthermore qPCR results revealed the *Atpds5a* mutation implies a complete inactivation of the gene. We also checked the relative expression levels of *AtPDS5A* in *Atpds5b* and of *AtPDS5B* in *Atpds5a*. The results obtained revealed that a mutation in one gene is not compensated by overexpression of the other one (Supplementary Figure S1). According to Genevestigator database, the transcription level of these genes is quite similar among the different tissues, and the expression of *AtPDS5B*, *AtPDS5C*, and *AtPDS5D* is slightly higher in the inflorescence. However, there are not noteworthy differences in the expression of the five genes throughout the distinct developmental stages (Hruz et al., 2008).

Homozygous plants for each insertion line grew normally and had no remarkable somatic abnormalities. *Atpds5a*, *Atpds5d*, and *Atpds5e* displayed normal fertility compared to WT plants, but *Atpds5b* and *Atpds5c* presented a slight reduction in the average seed set (**Table 1**). Mutant plants carrying single insertions in *AtPDS5A* and *AtPDS5B* were crossed and the corresponding double mutants were isolated in the F2 (*Atpds5a Atpds5b*). They were crossed with *Atpds5c* and afterward with *Atpds5e* to obtain triple (*Atpds5a Atpds5b Atpds5c*) and quadruple (*Atpds5a Atpds5b Atpds5c Atpds5e*) mutants. Unfortunately, we were unable to obtain the quintuple mutant because of the physical proximity of *AtPDS5B* and *AtPDS5D* (1.2 Mb; **Figure 1C**), and the progressive increase of developmental defects associated with the loss of AtPDS5 (**Figure 1**). We have only confirmed that the level of *AtPDS5D* expression does not vary in the quadruple mutant respect to the WT (Supplementary Figure S2). Double, triple and quadruple mutant plants germinated normally without any obvious delay in growth during the first 2 weeks. Later, they displayed markedly smaller rosette sizes and were much shorter and less robust than WT throughout their life cycle (**Figures 1D,E**). Mutants also displayed early flowering and reduced fertility. The majority of triple and quadruple mutant plants bolted 20 days after sowing whereas WT plants did so 30 days after sowing. The reduction in fertility was not only due to a decrease in seed set (**Table 1**) but also to the presence of short siliques (**Figure 1F**, Supplementary Table S3). However, there were not differences between *Atpds5a Atpds5b Atpds5c* and *Atpds5a Atpds5b Atpds5c Atpds5e* neither with respect to average seed set nor silique length (*p* = 0.10 and *p* = 0.37, respectively).

**Table 1:** Comparison between Col and *Atpds5* single, double, triple, and quadruple mutants respect to average seed set per silique.

	Seed set per silique	*p*-value
Col	51.53 ± 3.31	–
*Atpds5a*	54.33 ± 4.48	NS
*Atpds5b*	47.80 ± 3.73	^∗∗^
*Atpds5c*	44.53 ± 5.50	^∗∗∗^
*Atpds5d*	50.33 ± 4.47	NS
*Atpds5e*	47.67 ± 6.91	NS
*Atpds5a Atpds5b*	37.00 ± 3.59	^∗∗∗^
*Atpds5a Atpds5b Atpds5c*	30.27 ± 4.73	^∗∗∗^
*Atpds5a Atpds5b Atpds5c Atpds5e*	27.33 ± 4.84	^∗∗∗^

*Significance testing was carried out using the Student’s t-test. NS, not significant; ^∗∗^p < 0.01; ^∗∗∗^p < 0.001.*

### Male Meiosis Displays Chromosome Bridges at Anaphase I in the Different *Atpds5* Mutants

**FIGURE 2:**
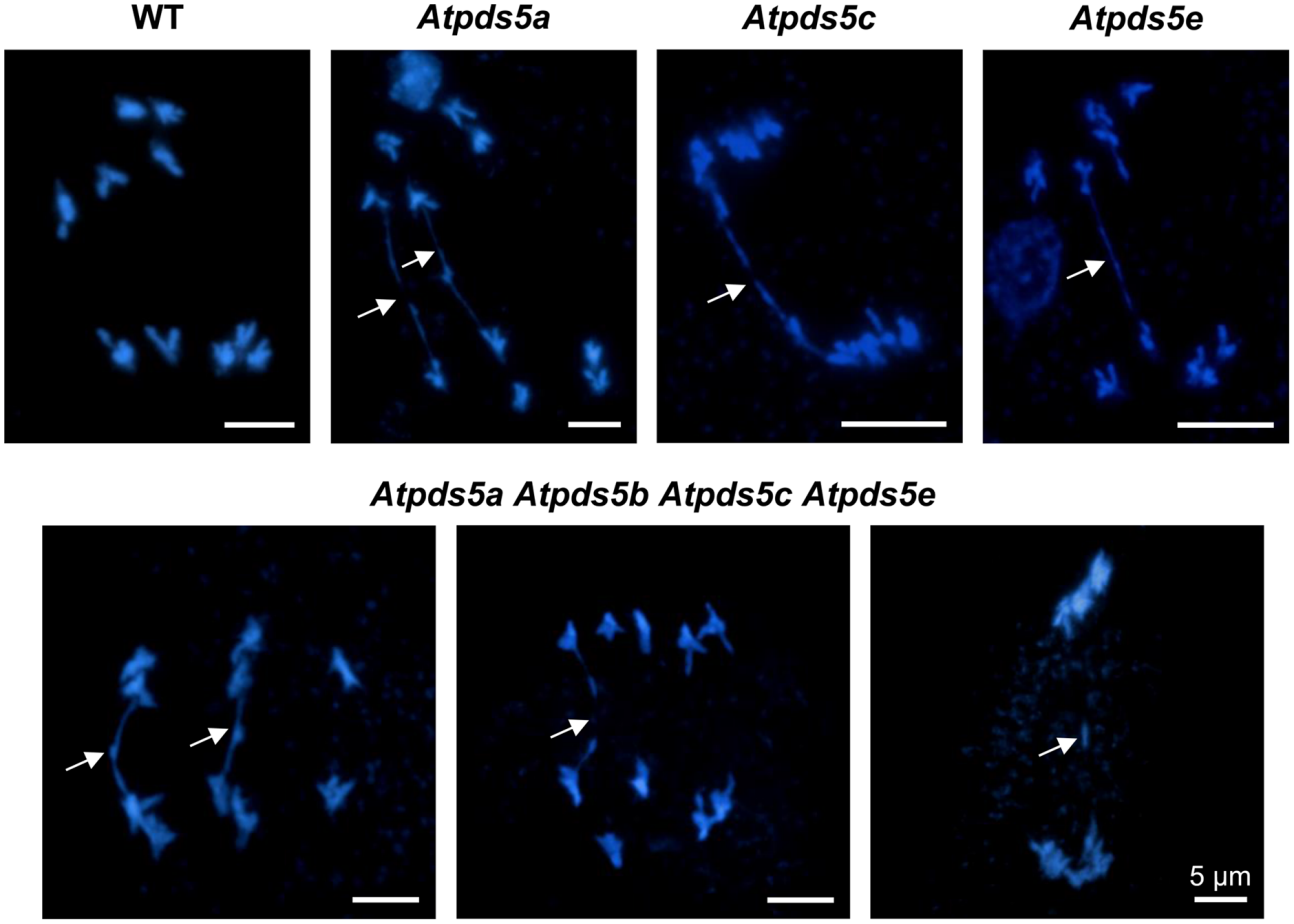
**Loss of AtPDS5 function generates chromosome bridges at anaphase I and telophase I.** Arrows indicate chromosome bridges, which probably arise because of the existence of unresolved recombination intermediates, and a fragment.

To determine whether meiosis defects could be responsible for the reduced fertility of *Atpds5* mutants, we analyzed DAPI-stained chromosome spreads from PMCs. Analysis of PMCs in *Atpds5* mutants revealed that meiosis proceeds without any important deviation from WT, even in the quadruple mutant (Supplementary Figures S3 and S4). We only detected, in contrast to the WT, the presence of some chromosome bridges at late anaphase I and telophase I in the single mutants, ranging from 20 to 25% (*n* = 40) (**Figure 2**). These bridges, originated probably as consequence of unresolved recombination intermediates, were also observed in the quadruple mutant with a similar frequency (22.5%; *n* = 40) and gave rise to fragments with a very low frequency (**Figure 2**).

Since sister chromatid cohesion is essential during meiotic recombination, we investigated whether the *Atpds5* mutants are affected in CO formation. We used 5S and 45S rDNA as FISH probes to identify each chromosome and chromosome arm of the complement set (Sánchez-Morán et al., 2002; Supplementary Figure S5). We only detected a significant decrease in the mean cell chiasma frequency of *Atpds5a Atpds5b Atpds5c Atpds5e* with respect to WT (9.32 ± 0.25 vs. 10.20 ± 0.14; *p* = 0.004). This difference was due to a slight reduction in the number of chiasmata in chromosome 1, specifically in its long arm (1.16 ± 0.09 vs. 1.54 ± 0.06; *p* = 0.001).

### AtSMC3 and SYN1 Localization Along Meiotic Chromosome Axes is Not Affected by the Absence of AtPDS5

**FIGURE 3:**
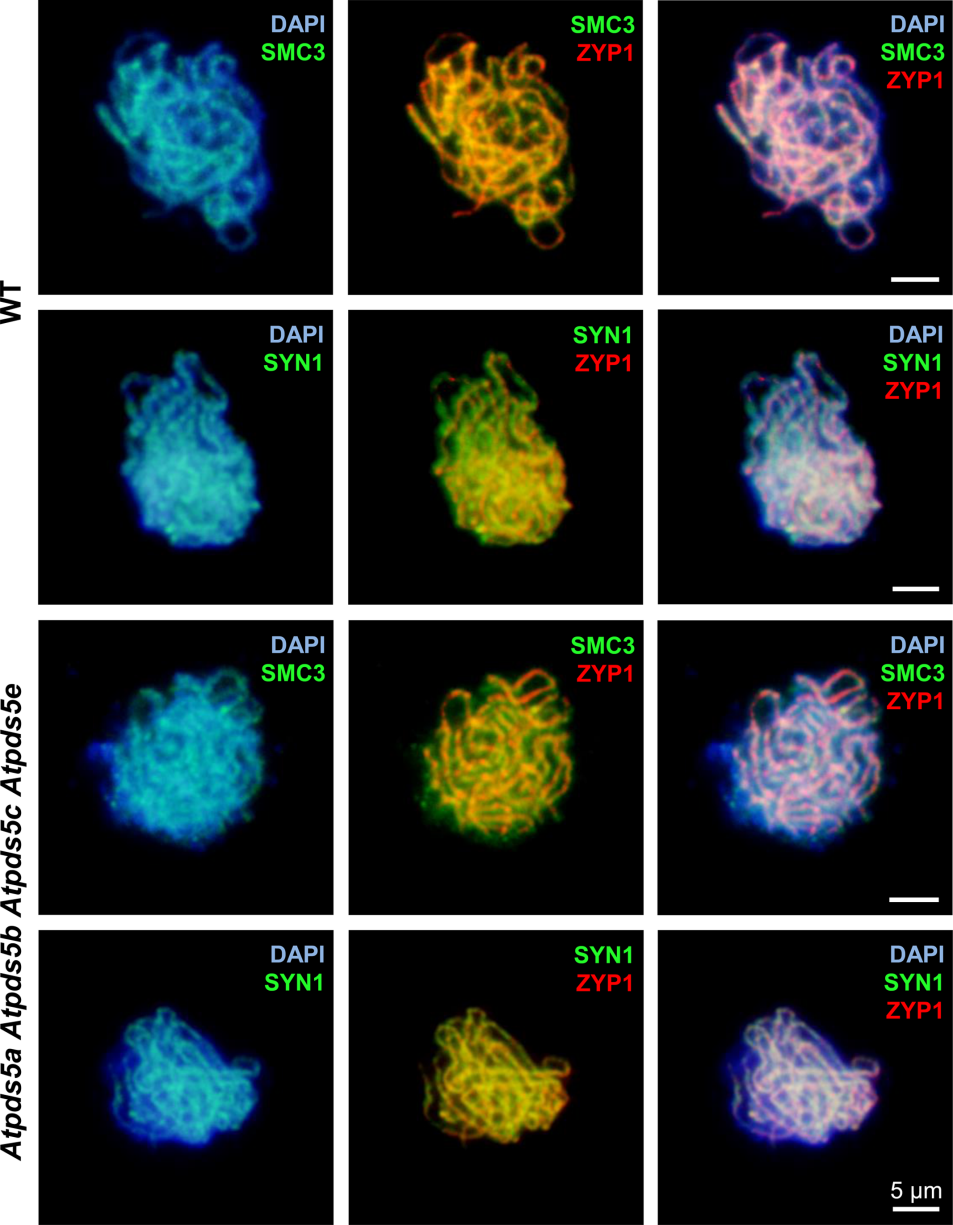
**Meiotic chromosome axes are normal in *Atpds5a Atpds5b Atpds5c Atpds5e* as revealed by immunolocalization of AtSMC3 and SYN1 at pachytene meiocytes**.

To further investigate any effect on chromosome axis morphogenesis, chromosome spread preparations of *Atpds5a Atpds5b Atpds5c Atpds5e* PMCs were examined by using anti-AtSMC3 and anti-SYN1 antibodies in conjunction with an antibody against the synaptonemal transverse filament protein, AtZYP1, to analyze the chronology of the early prophase I stages. AtZYP1 appears at early zygotene as short stretches which extend and produce a continuous signal between the synapsed homologous chromosomes at pachytene. AtSMC3 and SYN1 colocalize with chromosome axes during early prophase I. There were no obvious differences between WT and *Atpds5a Atpds5b Atpds5c Atpds5e* (*n* = 20) (**Figure 3**). This suggests that both proteins, AtSMC3 and SYN1, load normally on mutant chromosomes. Hence, AtPDS5 seems to be dispensable for meiotic cohesin complex formation.

### AtPDS5 Proteins Play a Role During DNA Repair

**FIGURE 4:**
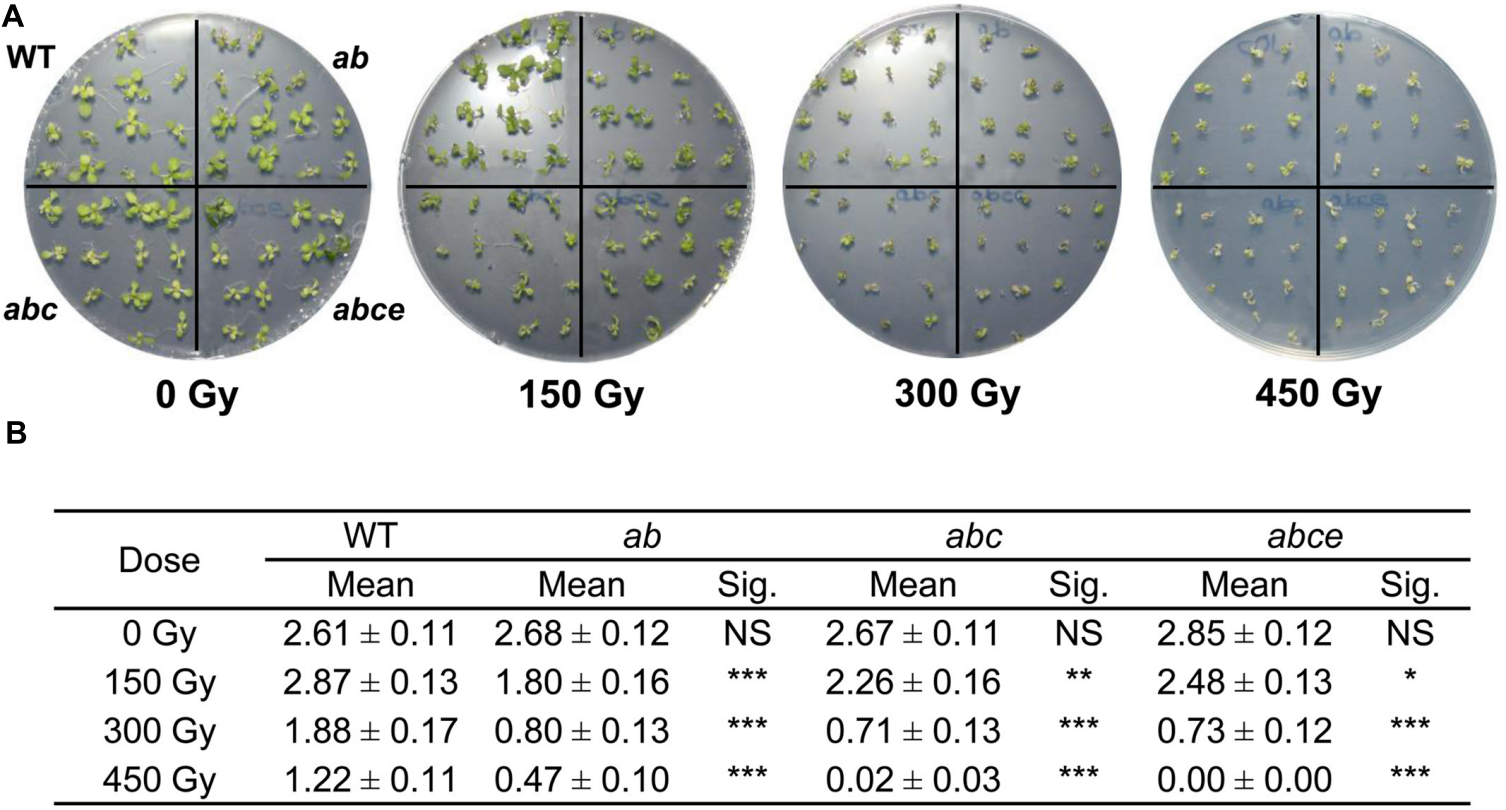
***Atpds5* double, triple, and quadruple mutants are hypersensitive to γ-rays. (A)** Phenotypes of 14-day-old seedlings (WT, double, triple, and quadruple mutants) after treatment with different radiation doses. **(B)** Mean number of true leaves per plant after treatment with different radiation doses. Mean values and standard errors are depicted. Asterisks indicate *p*-values from *t*-Student tests: NS, not significant; ^∗∗∗^*p* < 0.001, ^∗∗^*p* < 0.01, and ^∗^*p* < 0.05. *ab*: *Atpds5a Atpds5b*; *abc*: *Atpds5a Atpds5b Atpds5c*; *abce*: *Atpds5a Atpds5b Atpds5c Atpds5e*.

Many proteins involved in sister chromatid cohesion are important for the maintenance of genome integrity and repair of DNA damage during the cell cycle (da Costa-Nunes et al., 2006; Bolaños-Villegas et al., 2013). To assess whether AtPDS5 plays a similar role, we tested *Atpds5* mutants for hypersensitivity to γ-irradiation, a DSB-inducing agent, and the CL agents MMC, which mainly produces inter-strand CLs (Rink et al., 1996), and CDDP, which preferentially forms intra-strand CLs (Eastman, 1985; Boulikas and Vougiouka, 2003). Although both types of CL agents induce partially different types of DNA damage, both are expected to create DSBs during DNA synthesis, which are mostly repaired by HR. A hypersensitive response to γ-rays was consistently observed in the double, triple and quadruple mutants when compared with WT (**Figure 4**). Triple and quadruple mutants also showed hypersensitivity to MMC (Supplementary Figure S6). Finally, only the quadruple mutant revealed higher sensitivity than WT to high CDDP doses (Supplementary Figure S7). The global assessment of these results indicates that the quadruple mutant is more sensitive to DNA damage than the triple mutant, which in turn is more sensitive than the double mutant. Therefore, *AtPDS5* genes are involved in DNA damage response and the function of the different *AtPDS5* genes in DSB repair seems to be non-redundant.

### Alterations in the Expression of Several Genes Denote the Function of *AtPDS5* Genes in DNA Damage Response

**FIGURE 5:**
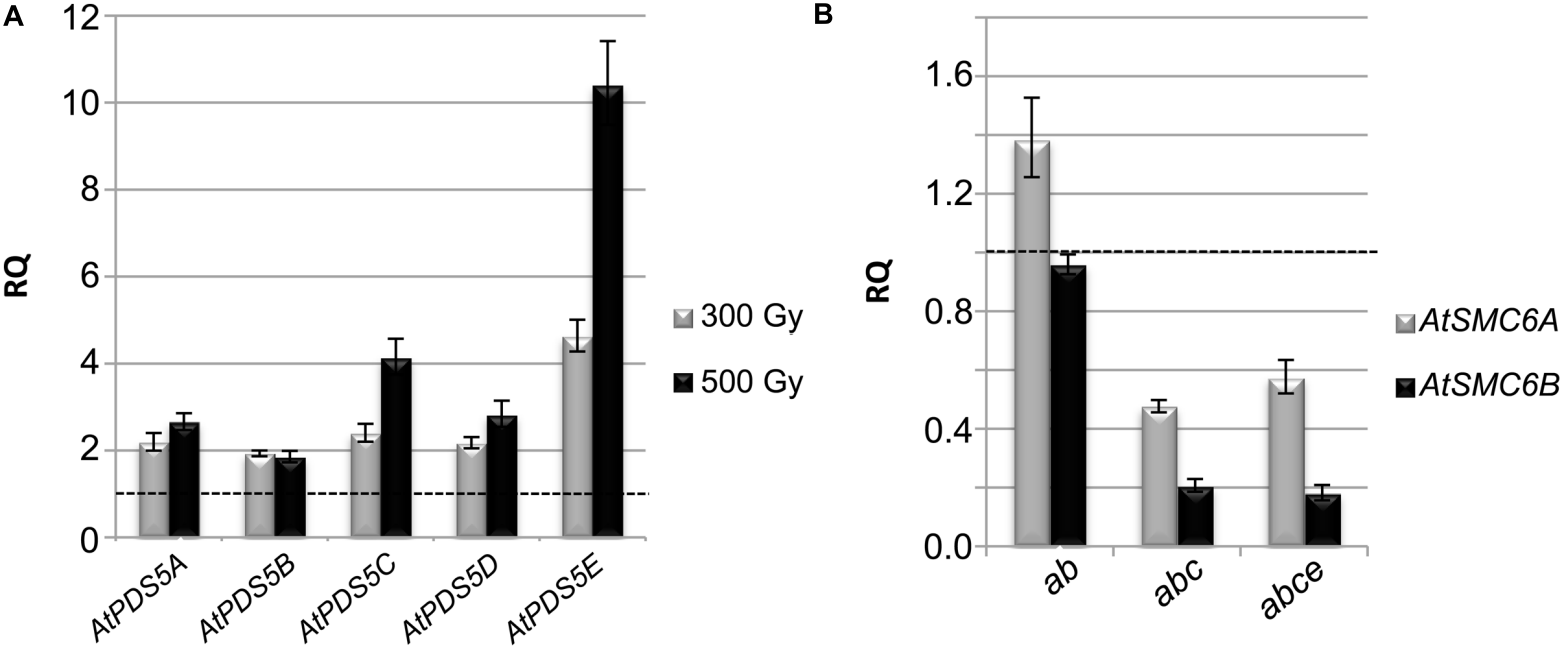
***AtPDS5* genes are involved in DNA repair, they are overexpressed after γ-irradiation and their loss of function generates down-regulation of *AtSMC6* genes. (A)** Expression analysis of *AtPDS5* genes after γ-irradiation in WT leaf samples. **(B)** Expression analysis of *AtSMC6A* and *AtSMC6B* in double, triple, and quadruple mutant leaf samples. Transcript levels are relative to non-irradiated WT (discontinuous line) (see Materials and Methods for more details). RQ: relative quantity. *ab*: *Atpds5a Atpds5b*; *abc*: *Atpds5a Atpds5b Atpds5c*; *abce*: *Atpds5a Atpds5b Atpds5c Atpds5e*.

Exposure of plants to DSB-inducing agents increases transcript levels of genes involved in DNA repair (Culligan et al., 2006). For this reason, we tested by qPCR the expression levels of the different *AtPDS5* genes after γ-irradiation treatment. The results obtained revealed increased levels in the expression of these genes at 300 and 500 Gy in leaf tissue (**Figure 5A**). The most noteworthy increase was observed in *AtPDS5E* at 500 Gy, the expression of this gene was 10-fold higher than in the untreated control. However, no significant change was observed for the expression of *AtPDS5* genes (except a slight increase for *AtPDS5E*) using samples from buds, containing meiocytes (Supplementary Figure S8A). We also investigated whether the loss of *AtPDS5* genes alters the expression of genes required for DNA repair, which includes the kinases *AtATM* and *AtATR*, *AtRAD50*, the ubiquitin ligase *AtBRCA1*, the recombinase *AtRAD51* and the SMC genes *AtSMC6A* and *AtSMC6B* (Gallego et al., 2001; Daoudal-Cotterell et al., 2002; Lafarge and Montané, 2003; Li et al., 2004; Culligan and Britt, 2008; Watanabe et al., 2009; Amiard et al., 2010; Pradillo et al., 2012). Small and unremarkable changes (down and up) were observed for *AtATR*, *AtRAD50*, *AtBRCA1*, and *AtRAD51* expression in leaf samples. *AtATM* exhibited a reduced expression in both the triple and the quadruple mutants and all these genes were down-regulated in bud samples (Supplementary Figure S9). However, the most significant result was observed for *AtSMC6A* and *AtSMC6B* since the transcripts of these genes in the triple and quadruple mutants were halved and reduced by more than half, respectively (**Figure 5B**). This reduction was also observed in bud samples (Supplementary Figure S8B).

### The Loss of *AtPDS5* Genes Affects Homologous Recombination in Somatic Cells

**FIGURE 6:**
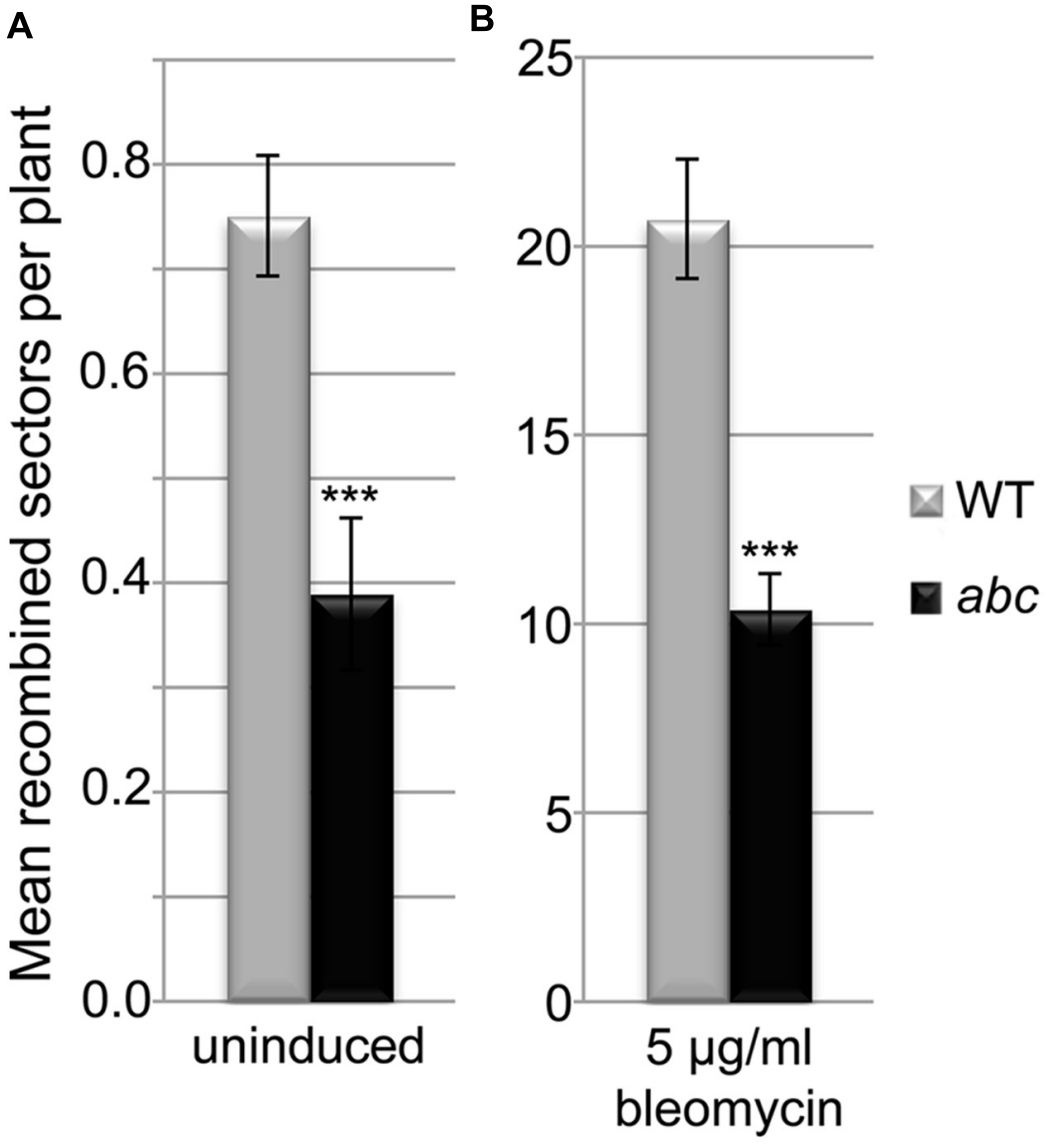
***AtPDS5* genes promote somatic homologous recombination.** Quantification of somatic HR events in WT and *Atpds5a Atpds5b Atpds5c* plants containing the *IC9* recombination reporter reveals a strongly significant reduction of HR frequency by about 50% in the triple mutant without **(A)** or with **(B)** treatment with 5 μg/ml bleomycin. Bars represent the mean number of recombination events per plant calculated from 4 replicates with 50 plants each. Error bars represent standard deviation. Statistical significance between WT and triple mutant was calculated using the Mann–Whitney test: ^∗∗∗^*p* < 0.001.

Taking into account the DNA repair defects and the alterations in the expression of several genes involved in DNA damage response observed, we decided to analyze a possible effect on somatic HR. As the T-DNA in *Atpds5e* contains a *GUS* gene that might interfere with the reporter system that is based on the restoration of this gene by HR, we concentrated our effort on the triple mutant *Atpds5a Atpds5b Atpds5c*. We crossed it with the reporter line *IC9* (Molinier et al., 2004). After isolation of the *Atpds5a Atpds5b Atpds5c IC9* line, we determined the HR frequency with and without induction of DSBs by bleomycin. In untreated plants, the WT IC9 control showed about 0.8 recombination events per plant, while the HR frequency in the *Atpds5a Atpds5b Atpds5c IC9* line was reduced by 50% to about 0.4 (**Figure 6A**; *p* < 0.001, *n* = 4, 50 plantlets each). Treatment with the DSB-inducing agent bleomycin induced the overall number of recombination events in both lines by a factor of 27. However, the *Atpds5a Atpds5b Atpds5c IC9* line still displayed a HR frequency that was only about 50% of the WT IC9 control line (**Figure 6B**; *p* < 0.001, *n* = 4, 50 plantlets each). Thus, HR efficiency is indeed reduced in the mutant background.

## Discussion

Cohesin cofactors are essential proteins during cohesin dynamics, although they are not components of the cohesin complexes. In this study we describe the role of one of these cofactors, PDS5, in *A. thaliana*, focusing on meiosis and DNA repair. This cofactor, together with WAPL, has been shown to play a role in the removal of cohesin from chromosomes (Sutani et al., 2009), although it is also required for the maintenance of cohesion by promoting a stable cohesin–chromosome interaction, at least in yeast (Vaur et al., 2012).

PDS5 is evolutionarily conserved from yeast to human and several studies have highlighted that it is required for both mitotic and meiotic divisions. Its role during meiosis is especially well known in *Sordaria*. In this species, SPO76/PDS5 is a chromosome structure component, which is axis-associated and assembles in association with axial element formation (van Heemst et al., 1999). The absence of this protein produces severe meiotic defects affecting chromosome morphogenesis (chromosomes are diffuse and kinky at midprophase), meiotic recombination, and sister chromatid cohesion (sister chromatids are fully separated at diplotene). Initiation of recombination in *spo76* nuclei seems to be normal, with high levels of RAD51 and DMC1 foci. However, these nuclei display a significant reduction in the number of late recombination nodules. In this situation, the defect in axial chromosome morphogenesis could affect recombination. On the other hand, axis destabilization in *spo76* is dependent on DSBs. Thus, SPO76 could enforce axis integrity in opposition to chromatin expansion forces produced by DSBs (Storlazzi et al., 2003, 2008). Zickler and colleagues have even proposed a meiosis-specific role for SPO76 since mitotic chromosomes in *spo76* mutants do not display sister chromatid cohesion defects. The role of this cohesin cofactor during meiosis has also been reported in other species. BIMD (*A. nidulans*), Pds5 (*S. cerevisiae*) and PDS5B (*M. musculus*) are also associated with meiotic chromosomes and required for normal chromosome compactness, playing an important role during meiosis (van Heemst et al., 2001; Jin et al., 2009; Fukuda and Hoog, 2010). Indeed, the absence of Pds5 in yeast produces SC formation between sister chromatids and not between homologs. This inter-sister SC formation requires the meiosis-specific cohesion subunit Rec8. Mice defective for REC8 also present inter-sister SC formation (Xu et al., 2005). Taken together, these data reveal that PDS5 family proteins are functional during meiosis, although with particular features in different species.

### Loss of AtPDS5 Proteins Does Not Affect Meiotic Chromosome Structure and Disturbs Slightly Meiotic Progression

In contrast to the species mentioned above, *A. thaliana* contains five *AtPDS5* genes. In this study we investigated their putative role during meiosis. The results obtained revealed that mutations in *ATPDS5B* and *ATPDS5C* produce a reduction in fertility. Curiously, and according to Genevestigator database, the expression of these genes is slightly higher in the inflorescence. Fertility defects are increased progressively when two, three or four AtPDS5 proteins are absent (**Table 1**, **Figure 1**). However, despite reduction in the average seed set, an exhaustive cytological examination of the meiotic process in PMCs of these mutants has not revealed apparent defects during this division (Supplementary Figures S3 and S4). Cohesion between sister chromatids appears mainly intact (**Figure 3**), as well as in yeast, in which Pds5 depletion does not affect chromosomal localization of Rec8 (Jin et al., 2009). Only *Atpds5a Atpds5b Atpds5c Atpds5e* showed a significant reduction in the mean cell chiasma frequency with respect to WT, because of a decrease in chromosome 1. Therefore, AtPDS5 proteins are not essential for chiasma formation. More noteworthy is the presence of chromatin bridges at anaphase I in all single mutants, which probably arise as a consequence of the existence of unresolved recombination intermediates (**Figure 2**). The frequency of these bridges is not increased in the quadruple mutant, suggesting all AtPDS5 proteins participate in release of sister chromatid cohesion during the first meiotic division.

On the other hand, according to the Bio-Analytic Resource for Plant Biology (BAR), the meiosis-specific cohesin SYN1 and AtSCC2, required for meiotic sister chromatid cohesion (Sebastian et al., 2009), are possible interactors of AtPDS5A. Moreover, topoisomerase AtTOPII and AtSUMO1 may interact with AtPDS5A and AtPDS5B. Interestingly, Topoisomerase II and SUMOylation of this protein have been revealed to be necessary for stress-relief along axis chromosomes during meiotic recombination in yeast (Zhang et al., 2014). Further analyses will be needed to determine a possible relationship between the function of AtTOPII and the presence of these chromatin bridges.

Unlike AtPDS5, other cohesin cofactors have been shown to play an essential role during *Arabidopsis* meiosis. *Atctf7* mutants, putative defective in AtSMC3 acetylation required for cohesion establishment, present defects in chromosome condensation and sister chromatid cohesion during male meiosis in addition to chromosome fragmentation. Furthermore, the localization of the cohesin complex subunits AtSMC3, SYN1 and AtSCC3 is diffuse and irregular during prophase I in some meiocytes of these mutants (Bolaños-Villegas et al., 2013; Singh et al., 2013). On the other hand, inactivation of the two *AtWAPL* genes also produces meiotic defects consisting of incomplete synapsis at pachytene, chromosome bridges at anaphase I and uneven nuclei at second meiotic division (De et al., 2014). In yeast, Ctf7 acetylation of Smc3 is critical for the establishment of cohesion by counteracting the Walp(Wpl1)-Pds5 complex (Rolef Ben-Shahar et al., 2008; Ünal et al., 2008; Rowland et al., 2009; Sutani et al., 2009), although recently it has been proposed that actually Wapl counteracts sister chromatid cohesion after it has been established. In addition, Wapl seems to be non-essential during meiotic chromosome segregation (Lopez-Serra et al., 2013). Thus, the function and interplay between AtWAPL and AtPDS5 seems to be different in *A. thaliana*, at least during meiosis. Obviously, a different result in complete absence of AtPDS5 function cannot be ruled out since we have only studied a situation in which four of the five genes are inactivated. However, the fact that a mutation in one *AtPDS5* gene is not compensated by overexpression of the others genes (Supplementary Figures S1 and S2) and the apparently absence of possible interactions identified between both cofactors (according to the BAR), suggest that AtWAPL and AtPDS5 play a different role in cohesion dynamics during *A. thaliana* meiosis, since AtWAPL is essential and AtPDS5 seems to have no (or little) impact on this division.

### AtPDS5 Proteins are Involved in Homologous Recombination During DNA Repair

Cohesins are essential proteins in the repair of DSBs. They facilitate DNA repair by holding sister chromatids together at the DSBs. Furthermore, apart from their genome-wide cohesion function, they have a direct role in DNA damage recognition and repair (Kim et al., 2002). The increase in the expression levels of the different *AtPDS5* genes we have found after γ-irradiation treatment may indicate their possible role in DSB repair (**Figure 5A**). Thus, the α-kleisin SYN2 (also known as AtRAD21.1) is also overexpressed after γ-irradiation (da Costa-Nunes et al., 2006). This protein has a specific function in DNA repair in *Arabidopsis* somatic cells and, unlike other cohesin complex subunits, its absence does not affect sister chromatid cohesion (Schubert et al., 2009). A similar function has also been recently described for SYN4 (AtRAD21.3), which has synergistic and non-redundant effect on the SYN2 function (da Costa-Nunes et al., 2014). We confirmed that the increase in *AtPDS5* transcripts is due to a specific role in DSB repair and not a consequence of a general deregulation produced by DNA damage by means of analyzing hypersensitivity to different DNA damage agents. We proved the quadruple mutant *Atpds5a Atpds5b Atpds5c Atpds5e* is hypersensitive to γ-rays, MMC and CDDP. We did not detect hypersensitivity to CDDP in the triple mutant *Atpds5a Atpds5b Atpds5c*, whereas the double mutant *Atpds5a Atpds5b* was only hypersensitive to γ-rays (**Figure 4**, Supplementary Figures S6 and S7). These findings suggest that AtPDS5 proteins share overlapping functions in DNA repair. Finally, we obtained more evidence for a specific role of *AtPDS5* genes in HR by analyzing blue sectors resulting from HR events affecting the *GUS* reporter gene. Results obtained in the HR assay reveal a strong reduction in the basic level of HR in somatic cells of the triple mutant *Atpds5a Atpds5b Atpds5c* respect to the WT. This mutant also exhibits a decrease in HR induction upon bleomycin treatment (**Figure 6**). Especially the reduced HR frequency after bleomycin treatment indicates a function of *AtPDS5* genes in DSB repair by HR. This might be a direct role in the regulation of cohesins at the site of a DSB. However, the same phenotype might also be explained by indirect effects of the *Atpds5a Atpds5b Atpds5c* mutations, e.g., the strong down-regulation of the expression of genes required for HR such as *AtSMC6* (for a detailed discussion see below).

The role of PDS5 in DNA repair has previously been described. *spo76-1* and *bimD6* mutants are sensitive to DNA-damaging agents (Moreau et al., 1985; Denison et al., 1993). In addition, the frequency of spontaneous mitotic interhomolog recombination is strongly reduced in *bimD6* (van Heemst et al., 2001). In *S. cerevisiae*, Pds5 is also involved in DNA repair and mutations in the gene produce accumulation in DNA breaks (Hartman et al., 2000; Ren et al., 2005). *S. pombe pds5* mutants are hypersensitive to both the alkylating agent methyl methanesulphonate (MMS) and bleomycin (Wang et al., 2002).

Contrary to the situation observed in meiosis, *Atpds5* mutants seem to be more similar to other *A. thaliana* mutants defective for cohesin related proteins involved in DNA repair. AtCTF7 is also required to DNA repair as revealed by comet assay after a bleomycin treatment and, as *Atpds5a Atpds5b Atpds5c Atpds5e* quadruple mutant*, Atctf7* also displays developmental defects (Bolaños-Villegas et al., 2013; Singh et al., 2013). However, results related to transcription expression levels of DNA repair genes are different since *AtATM*, *AtBRCA1* and *AtRAD51* are overexpressed in *Atctf7* with respect to WT (Bolaños-Villegas et al., 2013), whereas they do not in the quadruple *Atpds5* mutant or even show an underexpression as *AtATM*. Regarding AtWAPL, double mutants *Atwapl1 Atwapl2* do not display a dwarf phenotype. Nevertheless, the presence of chromosome bridges and chromosome fragments could indicate a role in DNA repair (De et al., 2014). Indeed, *wpl* yeast mutants are hypersensitive to DNA damaging agents (Game et al., 2003).

### AtPDS5 and the AtSMC5/AtSMC6 Complex

The architecture of the Smc5–Smc6 complex resembles that of the other SMC complexes. However, unlike cohesin, this complex is primarily required for DNA repair and mutations do not lead to premature chromatid separation (Torres-Rosell et al., 2005; Lindroos et al., 2006). Also, meiotic chromosome segregation and recombination are disturbed when the Smc5–Smc6 complex is dysfunctional in both fission and budding yeast (Pebernard et al., 2004; Farmer et al., 2011; Copsey et al., 2013). At present, no proper meiotic function has been described in *A. thaliana* for AtSMC5, AtSMC6A or AtSMC6B (there are two *AtSMC6* paralogs; Schubert, 2009). However, the complex is required for efficient repair by HR after DNA damage. *Atsmc5* homozygous mutants are lethal. *mim* mutants (defective for *AtSMC6B*) are sensitive to UV-C, X-rays, MMS and MMC (Mengiste et al., 1999). Furthermore, AtMSC6A and AtSMC6B are both necessary for the establishment of DSB-induced cohesion between sister chromatids to facilitate repair by HR. Indeed, recombination events, detected by scoring GUS-stained blue sectors, are drastically reduced in the single *Atsmc6* mutants, which are also defective in HR induction after bleomycin and MMC treatment (Watanabe et al., 2009). Therefore, the similarity between phenotypes corresponding to *Atsmc6* and *Atpds5* mutants could be related to the reduced expression of *AtSMC6* genes in the latter one (**Figure 5B**). We do not know whether the down-regulation of *AtSMC6* genes is a direct consequence of AtPDS5 failure, but the results suggest that the function of AtPDS5 might be related to the AtSMC5/AtSMC6 complex. In agreement with qPCR results discussed above, the expression pattern of *AtSMC6B* is different between *Atctf7* and *Atpds5a Atpds5b Atpds5c Atpds5e*, since this gene is overexpressed in *Atctf7* (Bolaños-Villegas et al., 2013).

In summary, the results presented here indicate that the AtPDS5 proteins are mainly involved in DNA repair, playing an important role during HR, and their function being very similar to that of the AtSMC5/AtSMC6 complex. Although we cannot rule out the possibility that a residual amount of AtPDS5 in the mutants analyzed was enough to ensure normal chromosome axis formation and accurate chromosome segregation, the role of this cohesin cofactor in *Arabidopsis* meiosis seems to be very different to that reported in other species since its significant decrease does not produce apparent cytological alterations. Further investigations will be needed to determine the biochemical relationships and the precise interplay between the different cohesin cofactors and SMC complexes. In this sense, obtaining a mutant without any functional AtPDS5 could shed light on this landscape. These studies will highlight similarities and differences between the involvement of these cofactors during mitosis and meiosis.

## Author Contributions

Conceived and designed the experiments: MP, AK, HP, JS. Performed the experiments: MP, AK, CO, JV, EC. Analyzed the data: MP, AK, CO, JV, HP, JS. Wrote the paper: MP, AK, HP, JS.

## Conflict of Interest Statement

The authors declare that the research was conducted in the absence of any commercial or financial relationships that could be construed as a potential conflict of interest.

## Footnotes

1 http://arabidopsis.info/

2 http://imed.med.ucm.es/Tools/sias.html

3 http://bar.utoronto.ca/welcome.htm

## Abbreviations

BAR: Bio-Analytic Resource for Plant Biology
CDDP: cisplatin
CL: cross-linking
CO: crossover
DMF,N: NDimethylformamide
DSB: Double-Strand Break
FISH: fluorescence in situ hybridization
GM: germination medium
HR: homologous recombination
MMC: mitomycin C
MMS: methyl methanesulphonate
NASC: Nottingham Arabidopsis Stock Centre
PDS5: Precocious Dissociation of Sisters 5
PMC: pollen mother cell
SC: synaptonemal complex
SIAS: sequences identities and similarities
SMC: structural maintenance of chromosome
WAPL: Wings Apart-Like
WT: wild-type
