# Supplementary material for: Involvement of the Cohesin Cofactor PDS5 (SPO76) During Meiosis and DNA Repair in Arabidopsis thaliana
Source: Front Plant Sci. 2015 Dec 1;6:1034. doi: 10.3389/fpls.2015.01034 (PMC4664637; doi:10.3389/fpls.2015.01034)
Supplement: Supplementary file 1 [file Presentation_1.PDF]

Supplementary Material

Involvement of the cohesin cofactor PDS5 (SPO76) during meiosis and DNA repair in *Arabidopsis thaliana*

Mónica Pradillo[1,*], Alexander Knoll[2], Cecilia Oliver[1], Javier Varas[1], Eduardo Corredor[1], Holger Puchta[2] and Juan L. Santos[1]

[1]Departamento de Genética, Facultad de Biología, Universidad Complutense, Madrid, Spain
[2]Botanical Institute II, Karlsruhe Institute of Technology, Karlsruhe, Germany

*** Correspondence:** Mónica Pradillo: pradillo@bio.ucm.es

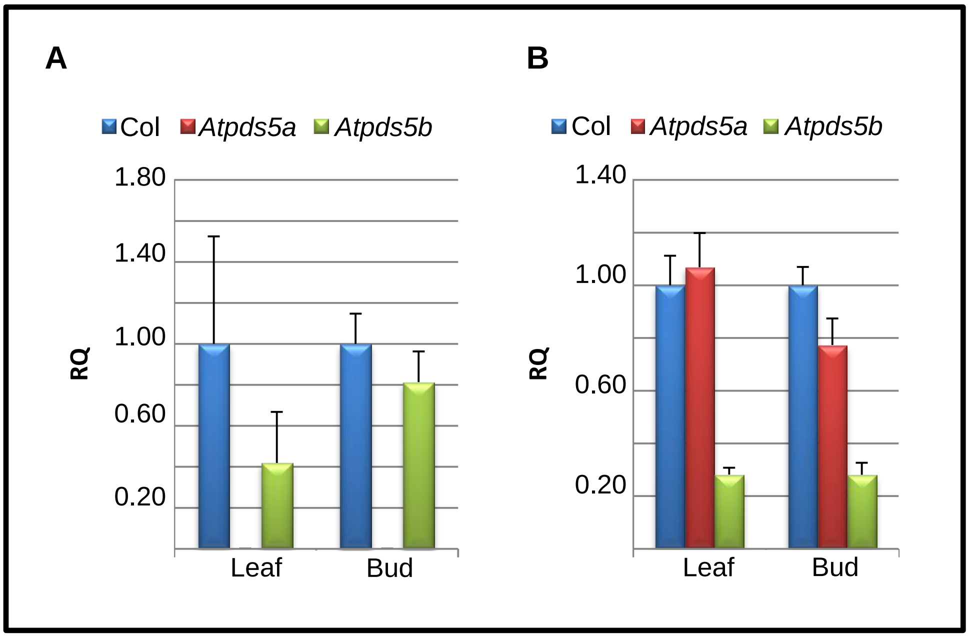

Supplementary Figure S1. Absence of either AtPDS5A or AtPDS5B is not compensated by overexpression of the other gene. (**A**) Expression analysis of *AtPDS5A* in *Atpds5a* and *Atpds5b* leaf and bud samples. *Atpds5a* mutation results in complete inactivation of the gene. (**B**) Expression analysis of *AtPDS5B* in *Atpds5a* and *Atpds5b* leaf and bud samples. Transcript levels are relative to WT (blue) (see Materials and Methods section for more details). RQ: relative quantity.

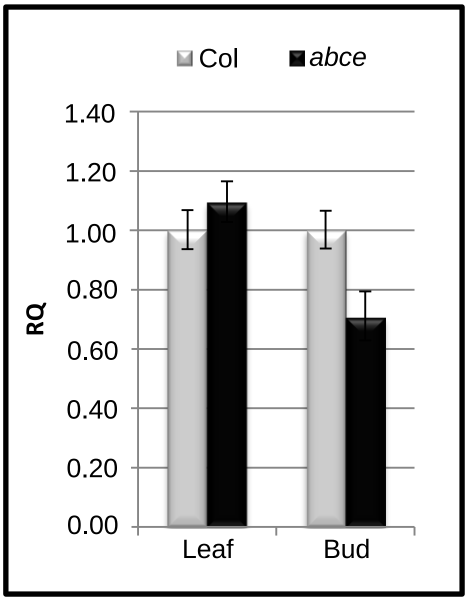

Supplementary Figure S2. *AtPDS5D* expression is not increased in the quadruple mutant *Atpds5a Atpds5b Atpds5c Atpds5e*. Expression analysis of *AtPDS5D* in *Atpds5a Atpds5b Atpds5c Atpds5e* leaf and bud samples. Transcript levels are relative to WT (grey) (see Materials and Methods section for more details). RQ: relative quantity.

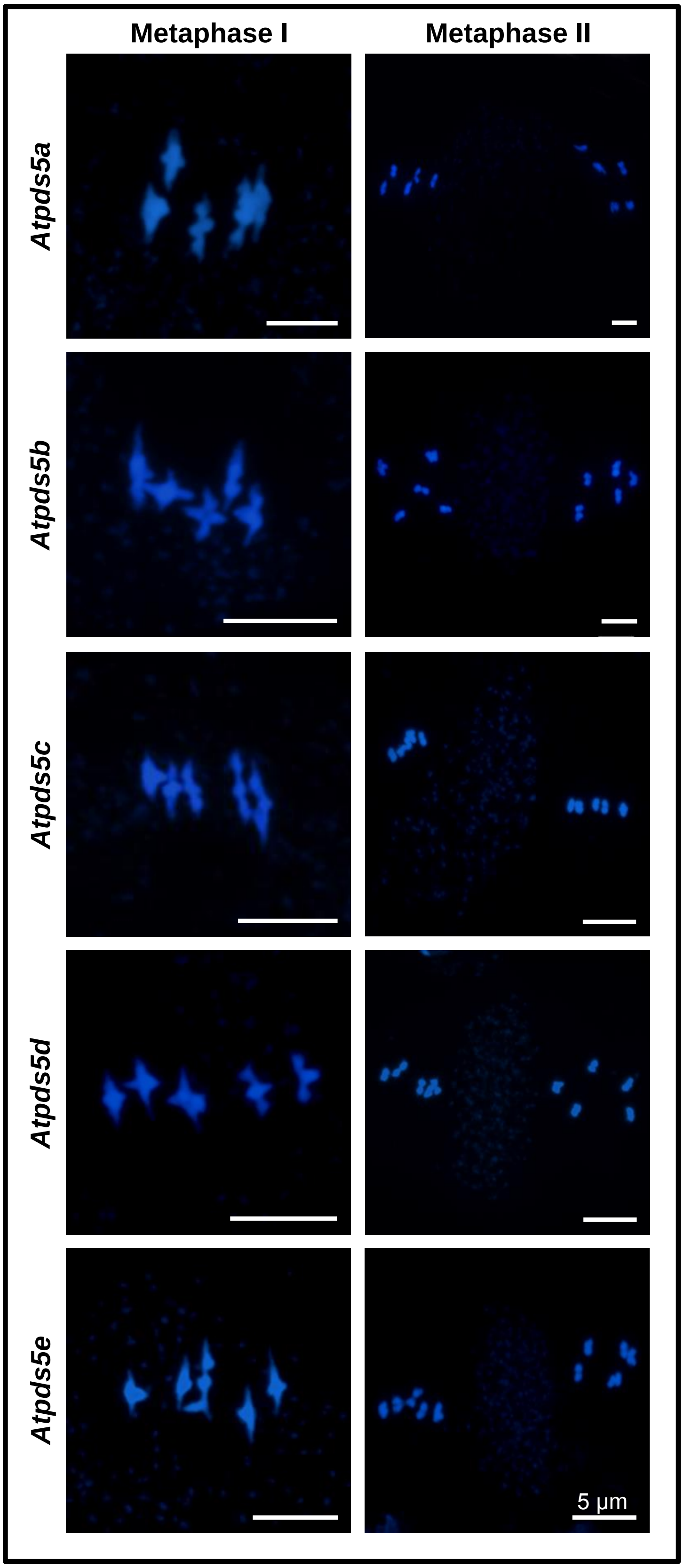

Supplementary Figure S3. Meiosis is normal in *Atpds5* single mutants.

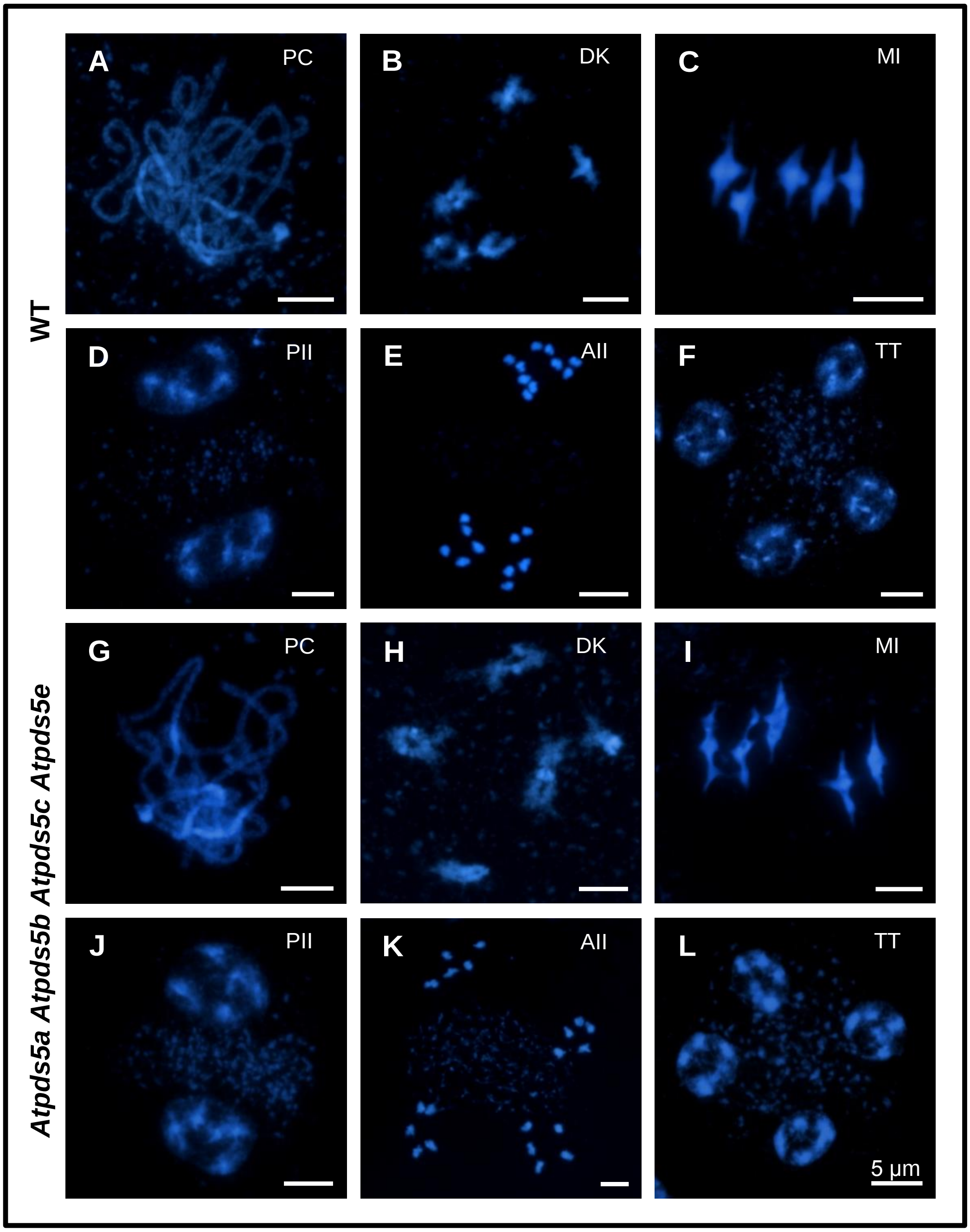

Supplementary Figure S4. *Atpds5a Atpds5b Atpds5c Atpds5e* does not display alterations during meiosis. DAPI stained chromosome spreads of PMCs at the first and second meiotic divisions. PC: Pachytene; DK: Diakinesis; MI: Metaphase I; PII: Prophase II; AII: Anaphase II; TT: Tetrad.

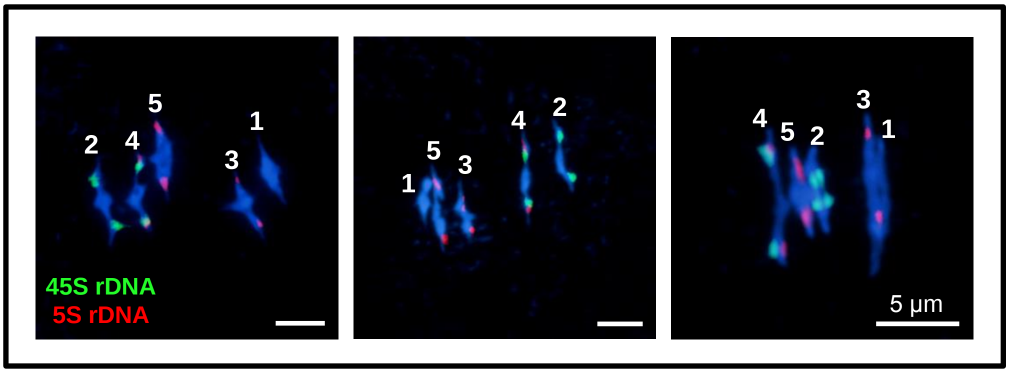

Supplementary Figure S5. *Atpds5a Atpds5b Atpds5c Atpds5e* just only presents a slight decrease in the mean cell chiasma frequency respect to WT, and five bivalents are always observed at metaphase I.

A

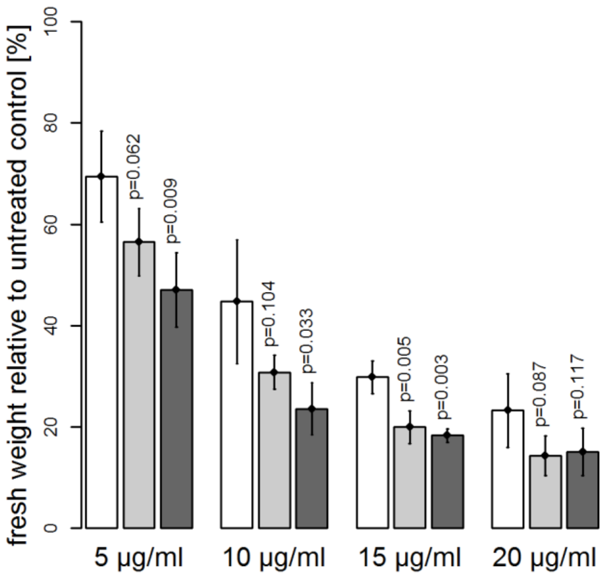

B

Dose	WT	*abc*		*abce*	
	Mean	Mean	Sig.	Mean	Sig.
5 µg/ml	69.43 ± 8.93	56.50 ± 6.60	NS	47.04 ± 7.37	**
10 µg/ml	44.75 ± 12.23	30.81 ± 3.30	NS	23.58 ± 5.13	*
15 µg/ml	29.83 ± 3.23	19.98 ± 3.24	**	18.33 ± 1.29	**
20 µg/ml	23.25 ± 7.25	14.30 ± 3.93	NS	15.12 ± 4.67	NS

Supplementary Figure S6. *AtPDS5* genes are involved in the repair of crosslinks induced by MMC. (**A**) The relative fresh weight of WT (white bars), *Atpds5a Atpds5b Atpds5c* (light grey bars) and *Atpds5a Atpds5b Atpds5c Atpds5e* (dark grey bars) plants treated with 5, 10, 15 and 20 µg/ml MMC is shown. At all concentrations but the highest, the quadruple mutant is significantly more sensitive than WT. There is no significant difference in relative fresh weight between triple and quadruple mutant lines. Bars represent the mean of relative fresh weights calculated from 4 replicates. Error bars represent standard deviation. The exact p-value is given above each line. (**B**) Mean relative fresh weights and standard deviations of wild-type, *Atpds5a Atpds5b Atpds5c* and *Atpds5a Atpds5b Atpds5c Atpds5e* plants treated with 5, 10, 15 and 20 µg/ml MMC. Statistical significance between WT and the triple or quadruple mutant, respectively, was calculated using the Mann-Whitney test: NS, no significance; ***p < 0.001, and **p < 0.01. *ab*: *Atpds5a Atpds5b*; *abc*: *Atpds5a Atpds5b Atpds5c*; *abce*: *Atpds5a Atpds5b Atpds5c Atpds5e*.

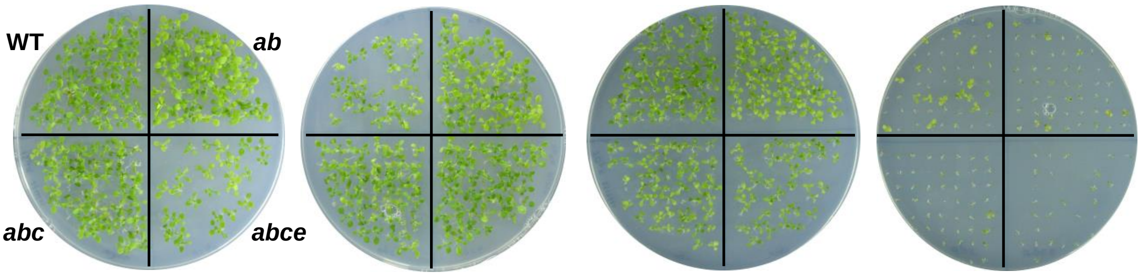

B

Dose	WT	*ab*		*abc*		*abce*	
	Mean	Mean	Sig.	Mean	Sig.	Mean	Sig.
0 µM	4.17 ± 0.06	4.39 ± 0.07	*	4.64 ± 0.09	***	3.96 ± 0.17	NS
15 µM	4.24 ± 0.07	4.40 ± 0.06	NS	4.38 ± 0.09	NS	3.87 ± 0.22	NS
30 µM	3.92 ± 0.05	3.90 ± 0.04	NS	3.95 ± 0.07	NS	3.68 ± 0.16	NS
50 µM	2.75 ± 0.08	2.75 ± 0.07	NS	2.61 ± 0.08	NS	2.45 ± 0.13	*
75 µM	0.32 ± 0.05	0.31 ± 0.05	NS	0.25 ± 0.05	NS	0.06 ± 0.06	***

Supplementary Figure S7. *Atpds5a Atpds5b Atpds5c Atpds5e* is hypersensitive to CCDP. (**A**) Phenotypes of 14-day-old seedlings (WT, double, triple and quadruple mutants) after treatment with different cisplatin doses. (**B**) Mean number of true leaves per plant after treatment with different cisplatin doses. Mean values and standard errors are depicted. Asterisks indicate p-values from t-Student tests: NS, no significance; ***$p < 0.001$, and *$p < 0.05$. *ab*: *Atpds5a Atpds5b*; *abc*: *Atpds5a Atpds5b Atpds5c*; *abce*: *Atpds5a Atpds5b Atpds5c Atpds5e*.

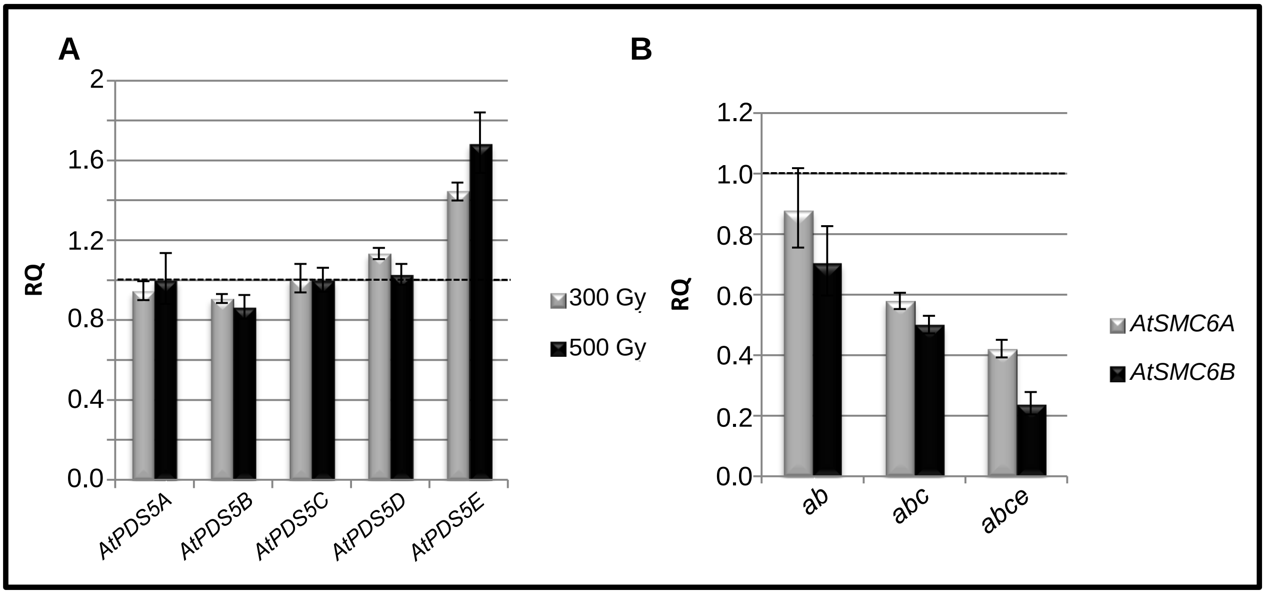

Supplementary Figure S8. In bud samples only *AtPDS5E* is overexpressed after γ-irradiation and loss of function of *AtPDS5* genes generates down-regulation of *AtSMC6* genes. (**A**) Expression analysis of *AtPDS5* genes after γ-irradiation in WT bud samples. (**B**) Expression analysis of *AtSMC6A* and *AtSMC6B* in double, triple and quadruple mutant bud samples. Transcript levels are relative to non-irradiated WT (discontinuous line) (see Materials and Methods section for more details). RQ: relative quantity. *ab*: *Atpds5a Atpds5b*; *abc*: *Atpds5a Atpds5b Atpds5c*; *abce*: *Atpds5a Atpds5b Atpds5c Atpds5e*.

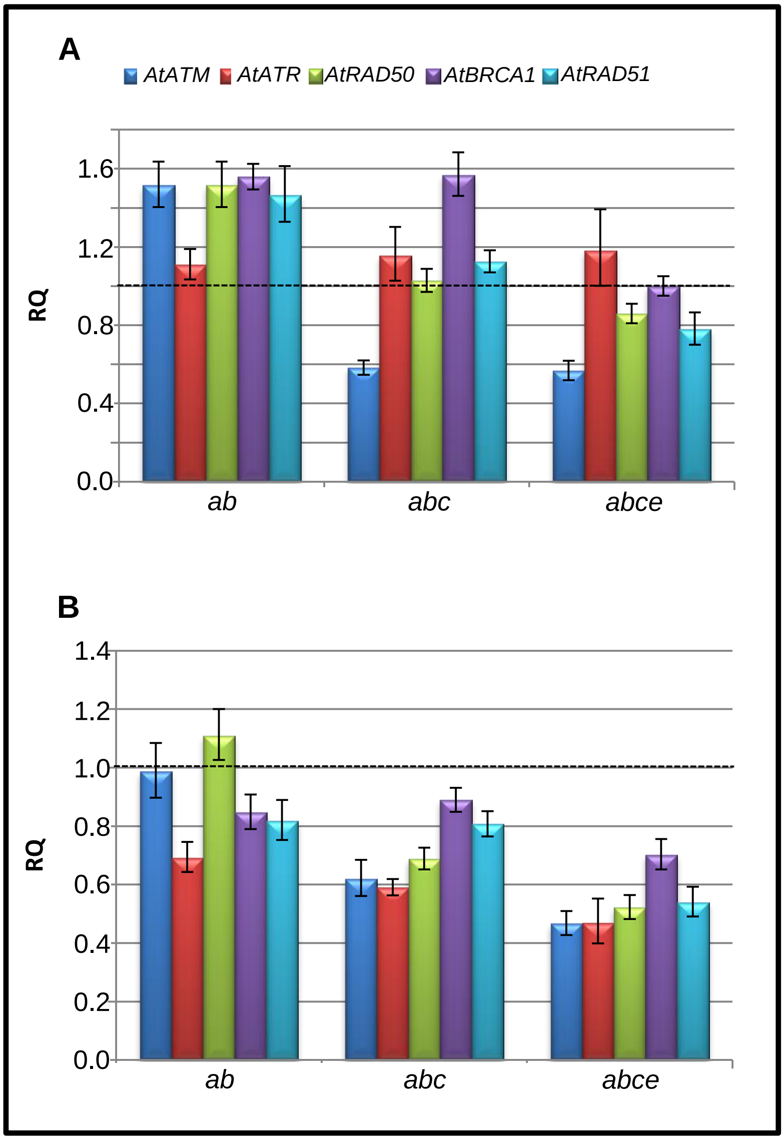

Supplementary Figure S9. *Atpds5* mutations produce *AtATM* down-regulation and alterations in the expression of several DNA repair genes.(**A**) Expression analysis of several DNA repair genes in double, triple and quadruple mutant leaf samples. (**B**) Expression analysis of several DNA repair genes in double, triple and quadruple mutant bud samples. Transcript levels are relative to WT (discontinuous line) (see Materials and Methods section for more details). RQ: relative quantity. *ab*: *Atpds5a Atpds5b*; *abc*: *Atpds5a Atpds5b Atpds5c*; *abce*: *Atpds5a Atpds5b Atpds5c Atpds5e*.

Supplementary Tables

Table S1. Primers used for plant genotyping and expression analysis (sequences from 5´ to 3´).

F: Forward primer; R: Reverse primer

	Primers for plant genotyping	Primers for expression analysis
Atpds5a-1	F: GAACTGCGCTATCTTCACCAG<br>R: ATGCTCACACTTCTATTGCGG	F: CAGCACCTGAAGCGCCTTACAG<br>R: CAGCATCTCTAACGTCACTTC
Atpds5b-1	F: ATCTCCTTAATTGCTCCCCAG<br>R: TGGGACCTAAATGATTCATAGAAC	F: ATCTCCTTAATTGCTCCCCAG<br>R: CCAGAACAGGGTAATAGTCACCG
Atpds5c-1	F: ATGTGAAGGGACATTCAGTGC<br>R: CATCAATGGCTTTCCCACGTC	F: GCAGTGAAATCGTCGGGTGTC<br>R: CTGTCCAGGAGTTCTGCAGGT
Atpds5d-1	F: TGTTGGATTTGACCAGCTTTC<br>R: AGAACCTTCTCCGCAAGACTC	F: ATGCAAAGTGCCCTAATTCCA<br>R: AGAACCTTCTCCGCAAGACTC
Atpds5e-1	F: GGTGCACTGAAGGCTGTAAGC<br>R: TGGATCAACCTCAAGCACCGT	F: GGTGCACTGAAGGCTGTAAGC<br>R: TGGATCAACCTCAAGCACCGT

Table S2. Primers and UPL probes used for qPCR (sequences from 5´ to 3´).

Gene	AGI code	Primers	UPL	Amplicon length (nt)
AtPDS5A	At5g47690	F: CTGAAGCGCCTTACAGTGATAAT<br>R: TTGCAACCGTTTCTAAAATGAG	134	129
AtPDS5B	At1g77600	F: AAAATTGACCCAGGAACTACTACTGA<br>R: GCTTAGGCTGCGCAAAAA	11	89
AtPDS5C	At4g31880	F: GGACAACTCATCAGCTAAAAAGG<br>R: CATCCTGGGACGTCTTGC	38	92
AtPDS5D	At1g80810	F: CTGCCAGAGTCCATTCCTTT<br>R: CACAGACACTGCCACGTTTT	34	78
AtPDS5E	At1g15940	F: TCAGCCAGTGAGGATAAGGAA<br>R: TCGGTTCCACATTCTTAGACAC	35	112
AtSMC6A	At5g07660	F: TGCCTCAAGATGCAACAAAC<br>R: AAAGTCGAGAAAGACCGTTCC	150	76
AtSMC6B	At5g61460	F: TCGCACGAGAGGATAAAGAAA<br>R: TGACTCAAAGCCGAGGATG	68	106
AtATM	At3g48190	F: AGGGTGGTGAGATGAGAAGC<br>R: TCTGTGTCAATTGCGTCTTGT	98	67
AtATR	At5g40820	F: TTCAGCGCCCAAAGAAGA<br>R: GGCTTGCAGAGGAATGGATA	3	67
AtRAD50	At2g31970	F: GCAGTGCAGGTCAAAAGGTT<br>R: GGCCCATCCAGGTTTGTAG	136	118
AtBRCA1	At4g21070	F: CCAAGAAATTGGTCTTATCTTGC<br>R: AGTTCCGCAAATTCTGCAAT	100	73
AtRAD51	At5g20850	F: CATGCCACCACAACAAGG<br>R: ACATGGCGAGCTTATCACTTTAC	91	78
ACT2	At3g18780	F: CCGCTCTTTCTTTCCAAGC<br>R: CCGGTACCATTGTCACACAC	30	78

F: Forward primer; R: Reverse primer

Table S3. Comparison between Col and *Atpds5* double, triple and quadruple mutants respect to silique length.

	**Silique length (mm)**	**p-value**
**Col**	12.47 ± 0.83	-
Atpds5a Atpds5b	9.80 ± 0.68	***
Atpds5a Atpds5b Atpds5c	7.67 ± 0.90	***
Atpds5a Atpds5b Atpds5c Atpds5e	7.93 ± 0.70	***

Significance testing was carried out using the Student's t-test.
NS, not significant; ***$p < 0.001$
